# The BMP2 Signaling Axis Promotes Invasive Differentiation of Human Trophoblasts

**DOI:** 10.3389/fcell.2021.607332

**Published:** 2021-02-04

**Authors:** Jiali You, Wei Wang, Hsun-Ming Chang, Yuyin Yi, Hongjin Zhao, Hua Zhu, Yu Sun, Minyue Tang, Chunyan Wang, Yimiao Sang, Guofang Feng, Shaobing Cheng, Peter C. K. Leung, Yi-Min Zhu

**Affiliations:** ^1^Department of Reproductive Endocrinology, School of Medicine, Women’s Hospital, Zhejiang University, Hangzhou, China; ^2^Department of Obstetrics and Gynaecology, BC Children’s Hospital Research Institute, University of British Columbia, Vancouver, BC, Canada; ^3^Department of Colorectal Surgery, School of Medicine, The First Affiliated Hospital, Zhejiang University, Hangzhou, China

**Keywords:** BMP2, early pregnancy loss, trophoblast invasion, trophoblast differentiation, embryo development, lncRNA

## Abstract

Embryo implantation and trophoblast invasion are principal limiting factors of pregnancy establishment. Aberrant embryo development or improper trophoblast differentiation and invasion may lead to various unfavorable pregnancy-related outcomes, including early pregnancy loss (EPL). Our clinical data show that the serum BMP2 levels were significantly increased during the first trimester of pregnancy and that the serum and BMP2 expression levels were lower in women with EPL than in women with normal early pregnancies. Moreover, we observed that BMP2 was expressed in oocytes and trophoblast cells of cleaved embryos and blastocysts prior to implantation in both humans and mice. Exogenous BMP2 promoted embryonic development by enhancing blastocyst formation and hatching in mice. LncRNA NR026833.1 was upregulated by BMP2 and promoted SNAIL expression by competitively binding to miR-502-5p. SNAIL induced MMP2 expression and promoted cell invasion in primary extravillous trophoblast cells. BMP2 promotes the invasive differentiation of mouse trophoblast stem cells by downregulating the expression of TS cell marker and upregulating the expression of trophoblast giant cell marker and labyrinthine/spongiotrophoblast marker. Our findings provide significant insights into the regulatory roles of BMP2 in the development of the placenta, which may give us a framework to explore new therapeutic strategies to pregnancy-related complications.

## Introduction

Implantation of a competent blastocyst into a receptive uterus is key for the establishment of pregnancy. Upon implantation, the outer monolayer of the blastocyst, consisting of trophectodermal (TE) cells, generates the first trophoblast lineages, which develop into diverse trophoblast cell types (Bianchi et al., 1993). Additionally, the inner cell mass (ICM) of the blastocyst develops into the second bilaminar extraembryonic tissue that gives rise to the embryo proper in mice and humans (Gardner, 1982; Stirparo et al., 2018). With the process of epithelial-mesenchymal transition (EMT), some TE cells develop into cytotrophoblast cells to form cell columns connecting to the endometrium. Extravillous trophoblasts (EVTs) are derived from cytotrophoblast cells in the anchoring columns under the influence of growth factors and cytokines derived from many cells, including decidual macrophages, uterine NK cells and stromal cells. After migration from the attached embryo, these highly invasive EVTs appropriately invade the uterine epithelium and uterine spiral arteries, a process that is indispensable for proper placentation and successful establishment of mammalian pregnancy (Wehrum et al., 2011; Gupta et al., 2016). In humans, the occurrence of EVT invasion in the endometrial stroma and myometrium (inner third) is critical for developing definitive maternal-fetal circulation and successful pregnancy (Cakmak and Taylor, 2011). Aberrant trophoblast (EVT or syncytiotrophoblast) differentiation or improper trophoblast invasion may lead to various unfavorable pregnancy-related outcomes, including early pregnancy loss (EPL), preeclampsia, intrauterine growth restriction, and choriocarcinoma (Brosens et al., 2011).

Given the complexity of embryo implantation and early placental development, it is likely that many mechanisms are involved in the pathophysiology of EPL. In the specimens obtained from EPL, the trophoblastic shell is thin and fragmented, and trophoblast infiltration around the lumen of the endometrial vessels and decidua is reduced. Placentation failure can be a primary event, as a result of a major chromosomal abnormality, or can be a secondary event as a result of early fetal demise due to a major developmental abnormality. Previous studies have shown that the degree of placentation defects and trophoblast apoptosis is increased in EPL, independent of the presence or absence of a chromosomal abnormality (Greenwold et al., 2003; Hempstock et al., 2003). Hence, a comprehensive understanding of the molecular mechanisms underlying trophoblast invasion is essential for improving the diagnosis and treatment of EPL.

As the largest subfamily of the transforming growth factor β (TGF-β) superfamily, bone morphogenetic proteins (BMPs) are essential promoting factors for organogenesis, including placental development (Shimasaki et al., 2004). Among the BMP members, BMP2 is detected in the murine endometrium during the period of decidualization and pregnancy establishment. The spatiotemporal expression of BMP2, which is correlated with mouse embryo implantation at the maternal-fetal interface, suggests that BMP2 plays an important role in the regulation of embryo implantation and early placentation (Ying and Zhao, 2000; Paria et al., 2001). In mice, conditional depletion of *Bmp2* in the uterus showed that the uterine stroma is incapable of undergoing decidualization to support further placental development, leading to sterility (Lee et al., 2007). Similarly, conditional depletion of the type II receptor for BMP2 (*Bmpr2*) in the mouse uterus resulted in fetal growth retardation and severe hemorrhage at the implantation sites, which subsequently caused fetal demise and placental abruption (Nagashima et al., 2013). Furthermore, the proinvasive effects of BMP2 have been reported in the EMT-related carcinogenesis of various cancers including breast, colon, gastric, and pancreatic cancers (Clement et al., 2005; Kang et al., 2010; Chen et al., 2011; Kim et al., 2015; Yang et al., 2015). Our previous studies revealed that BNP2 is expressed at a high level in primary human EVT cells and that BMP2 promotes the cell invasion of human trophoblast cells (Zhao et al., 2018a,b, 2020). Despite the essential role of BMP2 in the regulation of human trophoblast invasion, the precise molecular mechanisms by which BMP2 regulates trophoblast invasion remain largely unknown. Furthermore, the results obtained from conditional ablation of *Bmp2* in the uterus have indicated the critical role of BMP2 in regulating the transformation of the uterine stroma during embryo implantation in the mouse (Lee et al., 2007). However, it is unclear whether BMP2 is involved in the development of fetoplacental connections and the pathogenesis of EPL. The objective of this study was to use clinical samples and *in vitro* functional studies with human and mouse cells to investigate the expression, functional role and underlying molecular mechanisms of BMP2 in the regulation of trophoblast differentiation and invasion. Additionally, we aimed to obtain comprehensive information on the involvement of BMP2 in the pathogenesis of EPL (pregnancy loss at 5–8 weeks) and identify a therapeutic target for this pregnancy complication.

## Materials and Methods

### Subjects and Samples Collection

From October 2017 to April 2018, fifty healthy non-pregnant (Non-P) women were recruited as the control group; fifty pregnant women with a diagnosis of EPL attending Women’s Hospital, School of Medicine, Zhejiang University, Hangzhou, Zhejiang, China, were recruited as the study group. These pregnant women had vaginal bleeding and/or lower abdominal pain for the first time in the previous few days (0–2 days). The diagnosis of EPL was based on the clinical history, clinical examination, and transvaginal ultrasound (TVU) results. In cases where pregnancy structures (a gestational sac without fetal heart rate) were identified by TVU, the final diagnosis of EPL was made. Inclusion criteria were a gestational age between 5 and 8 weeks (based on the first day of the last menstrual period) and no history of recurrent spontaneous abortions, chromosomal abnormalities, endocrine diseases, anatomical abnormalities of the genital tract, infections, immunological diseases, trauma, internal diseases, or any chemical agent intake before their elective terminations; fifty age-matched women with a normal pregnancy (NP) who were undergoing terminations of pregnancy for psychological reasons at the same gestational age were designated the control pregnancy group. Blood samples were collected in EDTA-containing tubes (BD, Franklin Lakes, NJ, United States) and serum was isolated within 1 h by centrifugation at 1,900 × g for 10 min at 4°C to remove blood cells, and then at 16,000 × g for 10 min at 4°C to remove additional cellular nucleic acids attached to cell debris. Samples were stored at −80°C prior to analysis. Placental villous tissues were taken through the cervix during dilatation and aspiration according to strict clinical procedures. Embryos with arrested development or poor preimplantation morphology (zygotes, 4-cell stage, 8–16-cell stage, morulas, and blastocysts) that cannot to be used for transfer from assisted reproductive technology (IVF) patients were collected. Informed consent was obtained from each woman for the use of blood samples, placental villous tissue, and embryos, and the study was approved by the Ethical Review Committee of Women’s Hospital, Zhejiang University School of Medicine. All the samples were stored at −80°C or fixed in 4% formaldehyde until use.

### Measurement of BMP2 and MMP2 Activity

The BMP2 levels were quantified using a commercially available ELISA kit (Quantikine, BMP2 Immunoassay, R&D Systems, MN, United States). The MMP2 activity was measured using a commercially available ELISA kit (Quantikine, MMP2 Immunoassay, R&D Systems). All samples were assayed according to the manufacturer’s instructions and were tested in duplicate by personnel blinded to each patient group. The optical density of each well was determined using a microplate reader at an absorbance of 450 nm. No interference and no cross-reactivity were expected based on the manufacturer’s instructions. The minimum detectable dose (MDD) of BMP2 ELISA ranged from 4.3 to 29 pg/mL. The mean MDD was 11 pg/mL. The dynamic range of BMP2 ELISA ranged from 62.5 to 4,000 pg/mL. The mean MDD of MMP2 was 28.8 pg/mL. The dynamic range of MMP2 ELISA ranged from 28.2 to 43,070 pg/mL.

### First-Strand cDNA Synthesis and qPCR

Total RNA was extracted using TRIzol reagent (Life Technologies) following the manufacturer’s instructions. RNA concentrations were measured by absorbance at a 260 nm wavelength using a NanoDrop 2,000 Spectrophotometer (Thermo Fisher Scientific). Reverse transcription was performed using a QuantiTect Reverse Transcription Kit (QIAGEN), and 1 μg of total RNA was used according to the manufacturer’s instructions.

Real-time PCR was performed using the ABI Prism 7,300 sequencing detection system (PerkinElmer Applied Biosystems) in a 96-well microplate. In total, the 25 μl real-time PCR system contained 12.5 μl of SYBR Green qPCR MasterMix (Applied Biosystems), 7.5 μl of a diluted primer mixture (300 nM), and 5 μl of diluted cDNA template (25 ng RNA input). The primer sequences for real-time PCR are listed in Supplementary Data 1. The real-time PCR conditions were optimized as follows: 50°C for 2 min and 95°C for 10 min, followed by 40 cycles at 95°C for 15 s and 55°C for 1 min. The nucleotide sequences of the resultant PCR products were confirmed by sequencing. The relative mRNA expression levels were determined using the 2^–△^^△^^CT^ method. The mRNA and lncRNA expression levels were standardized to the endogenous GAPDH expression level.

### Western Blot Analysis

Cell extraction buffer (Life Technologies) was used to extract the entire cell lysate according to the manufacturer’s instructions. The protein lysates (30 μg) were electrophoresed on 8% SDS-polyacrylamide gels and transferred to a nitrocellulose membrane (Amersham Pharmacia Biotech). The membranes were immunoblotted with specific primary antibodies against BMP2, SNAIL, and MMP2 (listed in Supplementary Data 2) overnight at 4°C. The signals were detected with an enhanced chemiluminescence system (Amersham Pharmacia Biotech) after incubation with an HRP-conjugated secondary antibody (Santa Cruz Biotechnology). To standardize the levels of the protein loaded into each lane, the blots were reprobed with a polyclonal antibody directed against human α-tubulin. All primary antibodies used in this study are listed in Supplementary Data 2.

### Immunohistochemistry

Placental villous tissues were fixed in 4% formaldehyde and embedded in paraffin for sectioning. The placenta sections were deparaffinized and rehydrated before antigen retrieval with Dako antigen retrieval reagent (pH, 6.0). The sections were incubated with antibodies against BMP2 (1:50) at 4°C overnight following endogenous peroxidase blocking. A universal Dako-labeled streptavidin biotin-HRP system (Universal LSAB_Kit/HRP) was used for primary antibody detection. PBS containing rabbit IgG1 isotype (Abcam, ab172730, Cambridge, MA, United States) was used as a negative control and the concentration of the antibody used in IHC was 10 μg/ml. The sections were then exposed to a chromogen reaction (0.05% diaminobenzidine and 3% H_2_O_2_) and counterstained with Harris hematoxylin (Sigma). The signals were observed under a light microscope (Leica).

### Primary Human EVT Isolation and Culture

Thirty first-trimester human placentas (5–8 weeks gestation) were collected from women undergoing elective termination of pregnancy. Primary human EVT cells were isolated from chorionic villous explants as previously described and cultured at 37°C in a humidified 5% CO_2_/air atmosphere (Li et al., 2014). Briefly, the placenta villi tips were finely minced and cultured for 3–4 days in flasks with DMEM (Life Technologies) supplemented with 10% (vol/vol) FBS, 100 U/mL penicillin, and 100 μg/mL streptomycin. Non-attached pieces were removed and attached villous tissue fragments were cultured for another 10–14 days to allow for EVT outgrowth. EVT cells were subsequently separated from villous explants by trypsinization. Percoll gradient centrifugation, negative magnetic cell sorting using an antibody against classical major histocompatibility complex molecules and *in vitro* culture on a matrix-coated growth surface. The cells were fixed and probed with a specific antibody against cytokeratin-7 (EMD Millipore; MAB3554; Billerica, MA, United States) (10 μg/mL diluted at 1:100) followed by probing with a fluorescein isothiocyanate-conjugated secondary antibody or DAPI counterstaining. Next, we counted the number that was stained by cytokeratin-7 and DAPI. Only cultures showing more than 99% positive staining for cytokeratin-7 (Supplementary Figure 1) were used in this study. In general, cytotrophoblasts outgrow from villi onto Matrigel differentiate into EVT. Each experiment performed with primary EVT cells was replicated with cells from five different placentas. This study was approved by the Ethical Review Committee of Women’s Hospital, Zhejiang University School of Medicine, and all women provided informed written consent.

### Small-Interfering RNA Transfection

ON-TARGETplus siRNA (Thermo Fisher Scientific) targeting the target gene was transfected into primary EVTs or mouse trophoblast stem (TS) cells using Lipofectamine RNAiMAX Reagent (Life Technologies). The cells transfected with ON-TARGETplus control siRNA were used as a negative control in these studies.

### Matrigel-Coated Transwell Invasion Assay

Primary human EVT cell invasiveness was examined using the Corning Biocoat Growth Factor Reduced Matrigel Invasion Chamber (pore size, 8 μm; catalog no. 354483) according to the guidelines for use. Briefly, primary EVT cells were pretreated with vehicle control or BMP2 (25 ng/mL) for 20 min. Then, each insert was seeded with 5 × 10^4^ cells suspended in 250 μL vehicle/BMP2-containing DMEM supplemented with 0.1% (vol/vol) FBS, and 750 μL DMEM supplemented with 10% (vol/vol) FBS was added to the lower chamber. At the end of the experiment, non-invading cells were removed from the upper side of the membrane, and cells on the lower side were fixed in cold methanol for 20 min before they were stained with 0.1% crystal violet for 20 min. Membranes were cut out from the transwell inserts using a scalpel and mounted on glass slides with Cytoseal mounting medium. Cells on the lower aspect of the mounted membranes were viewed and photographed under a Nikon Eclipse 80i microscope. For quantification, the numbers of stained cells in five selected areas (top, middle, bottom, left, and right) were manually counted. Mean values from at least five independent experiments, each duplicated, were used in statistics.

### Luciferase Reporter Assay

Potential binding sites were predicted using the TargetScan database. The linear form of NR026833.1 or the 3′-UTR of SNAIL containing the binding site of miR-502-5p or mutated was cloned downstream of the Renilla luciferase gene in the dual luciferase plasmid pmirGLO vector (Promega) to construct the pmirGLO- NR026833.1 vector or pmirGLO- SNAIL-3′UTR vector. Briefly, EVT cells (1 × 10^5^ cells/well) were added to a 24-well plate for transfection. With the addition of 20 pmol of miR-502-5p mimics or negative control mimics, 0.8 μg of pmirGLO- NR026833.1 vector, pmirGLO-SNAIL-3′UTR vector or pmirGLO vector, were co-transfected into cells using Lipoimax (Invitrogen). Overexpression of miR-502-5p mimics after transfection in the cells was assessed by qPCR. After transfection for 48 h, the cells were harvested, and luciferase activities were measured using the Dual-Luciferase Reporter Assay System (Promega, Madison, WI, United States). Luciferase activity was measured as firefly luciferase/renilla luciferase ratio.

### Oligonucleotide Transfection, miR Assay, and lncRNA Assay

The transient transfection was carried out when the cultured cells reached 60–70% confluence. si-RNA, si-LncRNA, miRNA mimic and their related control oligonucleotide were designed and synthesized by RiboBio (Guangzhou, China). All the transfection procedures were performed using the final concentration of 60 nM of miRNA mimics, 100 nM of miRNA inhibitor or si-LncRNA. LipoRNAi Max (Invitrogen, Carlsbad, CA, United States) was used as the transfection medium according to the manufacturer’s instructions.

### Superovulation and Embryo Collection

Female ICR mice (6–8 weeks; Shanghai SLAC Laboratory Animal Co., Ltd., Shanghai, China) were intraperitoneally injected with 10 IU of pregnant mare serum gonadotropin (PMSG; Ningbo Second Hormone Factory, Ningbo, China), followed by a 10 IU injection of human chorionic gonadotropin (hCG; Ningbo Second Hormone Factory, Ningbo, China) 48 h after PMSG injection. Female mice were mated with male ICR mice (8–10 weeks; Shanghai SLAC Laboratory Animal Co., Ltd.) after the hCG injection. Mouse embryos were collected from female ICR mice if a vaginal plug was present. A total of 30 female mice were used for superovulation and three mice were used in each stage of the immunofluorescence experiment (a total of 21 female mice). The processes of blastocyst formation and hatching were observed using microscopy. Six independent experiments (*n* = 6) were performed, resulting in a total of 110 embryos in the control group and 111 embryos in the BMP2-treatment group (a total of 9 female mice). Unfertilized oocytes were collected from the ampulla of the oviducts at 14 h after hCG injection without mating. Zygotes were obtained from the ampulla of the oviducts of plug-positive females at 18 h post-hCG injection. Cumulus cells were dispersed with 0.3 mg/mL hyaluronidase. Other preimplantation mouse embryos were collected at 42–45 h (the 2-cell stage), 52–55 h (the 4-cell stage), 65–68 h (the 8-cell stage), and 93–96 h (the blastocyst stage) after hCG injection. Embryos were flushed from the oviducts and uterus using M2 medium (M7167, Sigma-Aldrich). All experimental procedures were performed in accordance with the guidelines of the Guide for the Care and Use of Laboratory Animals and approved by the Animal Ethics Committee of Zhejiang University for animal experiments.

### Embryo Immunofluorescence

The embryos were washed three times with phosphate-buffered saline (PBS) containing 0.5% bovine serum albumin (BSA, Sigma-Aldrich) and fixed in 4% paraformaldehyde for 30 min. Fixed embryos were washed three times in PBS containing 0.5% BSA, used immediately, or stored at 4°C in embryo storage buffer (PBS + 0.9% sodium azide) for up to 1 week. Fixed embryos were permeabilized with 0.01% Triton X-100 for 30 min and washed three times with PBS containing 0.5% BSA before being blocked in a 5% BSA/PBS solution for 1 h. Embryos were incubated with a primary antibody against BMP2 at a 1:500 dilution in 5% BSA/PBS overnight at 4°C. Finally, these embryos were washed three times for 20 min in 0.5% BSA/PBS containing 0.05% Tween 20 (0.5% BSA/PBST) and incubated with a fluorescein isothiocyanate-conjugated secondary antibody (1:200, Invitrogen, Carlsbad, CA, United States) for 1 h. The nuclei were stained with 1 mg/mL 40′, 6′-diamidino-2-phenylindole (DAPI, Sigma-Aldrich) for 10 min. Embryos at the 8-cell stage were chosen as negative controls. Images were taken on an Olympus FV1000 confocal microscope and processed using Adobe Photoshop. The same experiment was independently repeated three times, each time in triplicate, and 20–30 embryos of different developmental stages were examined each time.

### Embryo Culture

To study the role of BMP2 in preimplantation embryo development and zona hatching (complete escape of the blastocyst from its zona pellucida) *in vitro*, two-cell embryos on day 2 (08:30–09:00 h) were recovered and pooled from several mice in M2 medium (M7167, Sigma-Aldrich). The embryos were washed three times in KSOM medium. Embryos were cultured in groups in microdrops (50 μl) of Whitten’s medium under light oil in an atmosphere of 5% CO_2_/95% air at 37°C for 72 h in the presence or absence of BMP2 (100 ng/ml). BMP2 was added when the cultures were started. The embryos were observed every 24 h to monitor their development. Each experiment was repeated six times with the exception of numerous replicates of controls included in each experimental repetition.

### Mouse TS Cell Culture and Differentiation Induction

The mouse TS cells line was kindly donated by Dr. Haibin Wang from the Chinese Academy of Sciences (Beijing, China). Mouse trophoblast stem cells (TS cells) that were established from mouse 3.5 days postcoitum blastocysts or the extraembryonic ectoderms of 6.5 days postcoitum embryos can either self-renew or differentiate into distinct trophoblastic cell populations, including invasive mouse trophoblastic giant cells (Tanaka et al., 1998; Uy et al., 2002). Briefly, TS cells were maintained and propagated in 25 ng/mL fibroblast growth factor 4 (FGF4) culture medium supplemented with 1 mg/mL heparin (Sigma-Aldrich) and composed of 30% TS medium (RPMI 1640 supplemented with 20% FBS, 50 mg/mL penicillin/streptomycin, 2 mM L-glutamine, 1 mM sodium pyruvate, and additional additives, including 50 mM β-mercaptoethanol) and 70% mouse embryonic fibroblast (MEF) conditioned medium. Differentiation of the mouse TS cells was induced by the removal of FGF4, heparin, and MEF-conditioned medium for 7 days.

### Statistical Analysis

All statistical analyses were performed using SPSS 16.0 (SPSS, Chicago, IL, United States) and GraphPad Prism 5.0 (GraphPad Software, Inc., San Diego, CA, United States). Data are presented as the mean ± SD from at least five independent experiments. Differences between groups were determined by Student’s *t*-test or one-way analysis of variance, and statistical significance was defined as *P* < 0.05. Differences in the rates of development and hatching between the protease treatment groups and the control group were analyzed by a χ^2^-test. *P* < 0.05 was defined as statistically significant.

## Results

### Decreased Serum Levels of BMP2 Are Associated With EPL

**FIGURE 1:**
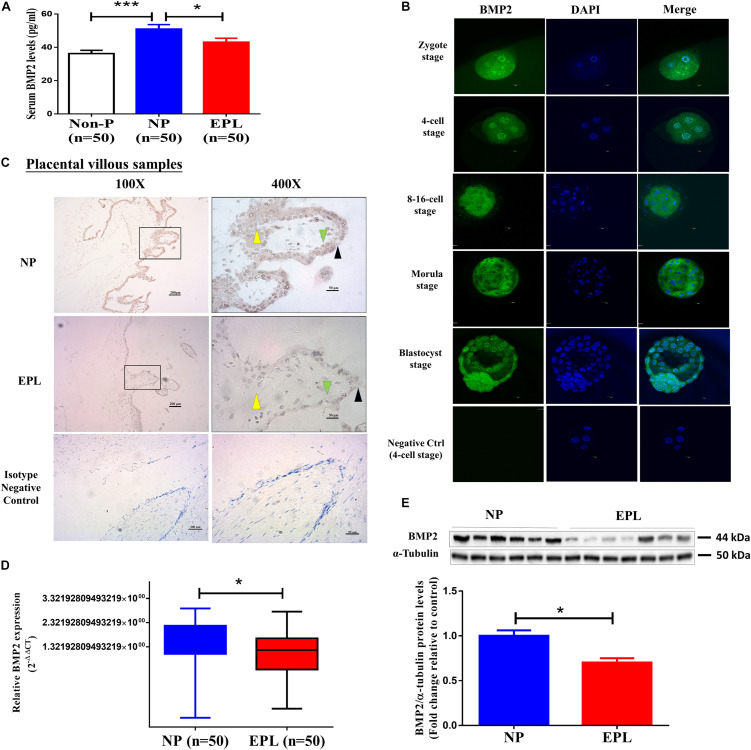
BMP2 expression in women with normal early pregnancies and EPL. **(A)** Comparison of the circulating BMP2 levels in non-pregnant (Non-P) women (*n* = 50), normal early pregnant women (NP, *n* = 50) and women with EPL (*n* = 50). The serum samples collected from these women were measured using ELISA. **(B)** The expression and localization of BMP2 in human preimplantation embryos. Human preimplantation embryos, including the zygote, 4-cell, 8-16-cell, morula and blastocyst stages, were fixed and probed with a specific antibody against human BMP2 followed by probing with a fluorescein isothiocyanate-conjugated secondary antibody or DAPI counterstaining. PBS without primary antibody was used as a negative control. The images were detected using confocal microscopy. **(C)** Expression and localization of BMP2 in first-trimester human placental villus tissues obtained from women with NPs and EPL. The images were detected using immunohistochemistry staining. PBS containing rabbit IgG1 isotype was used as a negative control. Stronger brown cytoplasmic staining of BMP2 was detected in the cell column EVTs (yellow arrowheads), syncytiotrophoblasts (black arrowheads) and CTBs (green arrowheads) of the placental villous tissues obtained from women with NPs (scale bar 1/4 200 mm in 100 × images and scale bar 1/4 50 mm in 400 × images). **(D,E)** The mRNA **(D)** and protein **(E)** levels of BMP2 in villous tissue obtained from women with NPs (*n* = 50) and EPL (*n* = 50) were examined using RT-qPCR and western blot analyses, respectively. The relative amounts of mRNA were calculated using the 2^–ΔΔ*Ct*^ method and normalized to that of the internal control gene *GAPDH*. The relative amounts of protein were normalized to that of the internal control α-tubulin. Differences between groups were determined by either Student’s *t*-test or one-way analysis of variance (*P < 0.05, ***P < 0.001).

To investigate the functional role of BMP2 during early pregnancy in humans, we first examined the serum levels of BMP2 by recruiting three groups of women, including healthy non-pregnant (Non-P) women, women with NP and women with EPL. The clinical and biochemical characteristics of the women included in this study are shown in Table 1. Women in all three groups (*n* = 50 in each group) were of similar age. Women in the NP group had a similar gestational age to those in the EPL group (47.06 ± 4.99 days vs. 48.76 ± 4.18 days, respectively, *P* > 0.05). Maternal serum β-HCG levels were significantly higher in the NP group than in the EPL group (38,427.18 ± 41,159.79 IU/mL vs. 22,510.28 ± 30,457.91 IU/mL, respectively, *P* < 0.05). Compared with Non-P women, women with NPs had significantly higher serum BMP2 levels (36.28 ± 13.82 pg/mL vs. 51.02 ± 18.77 pg/mL, respectively, *P* < 0.001) (Table 1 and Figure 1A). Notably, subjects in the NP group had higher serum BMP2 levels than those in the EPL group (51.02 ± 18.77 pg/mL vs. 43.16 ± 16.35 pg/mL, respectively, *P* < 0.05) (Table 1 and Figure 1A).

**TABLE 1:** Clinical and biochemical characteristics of the women included in this study.

	**Non-pregnacy (Non-P, *n* = 50)**	**Normal pregnancy (NP, *n* = 50)**	**Early pregnancy loss (EPL, *n* = 50)**
Age (year)	28.04 ± 3.14	28.06 ± 4.65	28.92 ± 3.48
Gestational age (day)	−	47.06 ± 4.99	48.76 ± 4.18
Serum β-HCG (IU/mL)	−	38,427.18 ± 41,159.79^a^	22,510.28 ± 30,457.91^a^
Serum BMP2 (pg/mL)	36.28 ± 13.82^b^	51.02 ± 18.77^bc^	43.16 ± 16.35^c^

*Data are presented as the means ± SD. ^*a*^P < 0.05; ^*b*^P < 0.001; ^*c*^P < 0.05.*

### The Expression of BMP2 Is Higher in Women With Normal Early Pregnancies Than in Those With EPL

Although the expression of BMP2 at the maternal-fetal interface has been reported (Paria et al., 2001), whether BMP2 is expressed in human embryos remains to be elucidated. We thus used immunostaining analysis to examine the expression and localization of BMP2 in human preimplantation embryos. As shown in Figure 1B, immunostaining for BMP2 was observed in all embryos analyzed at different stages of development. At the zygote and 4-cell stages, BMP2 was mainly expressed around the nucleus, and BMP2 was mainly localized in the cytoplasm from the 8-cell stage of embryos. Notably, strong cytoplasmic BMP2 staining was observed in the trophectoderm, the cell layer from which the trophoblast differentiates (Figure 1B). Nonetheless, BMP2 was also expressed in the ICM that gives rise to the definitive structures of the fetus (Figure 1B). Given that the serum BMP2 levels were decreased in women with EPL, we next compared the expression of BMP2 in placental villous tissues obtained from women with NPs and those from women with EPL using immunohistochemistry. As shown in Figure 1C, the results revealed that the immunohistochemical staining for BMP2 was significantly decreased in the cytoplasm of cell column EVT, syncytiotrophoblastic (synCTB) and cytotrophoblastic (CTB) cells in the villous tissues obtained from women with EPL. Similarly, the mRNA and protein levels of BMP2 were reduced in cell lysates of the villous tissues obtained from women with EPL (Figures 1D,E and Supplementary Figure 2).

### BMP2 Upregulates MMP2 Expression, Increases MMP2 Activity and Promotes Cell Invasion in Primary Human EVT Cells

**FIGURE 2:**
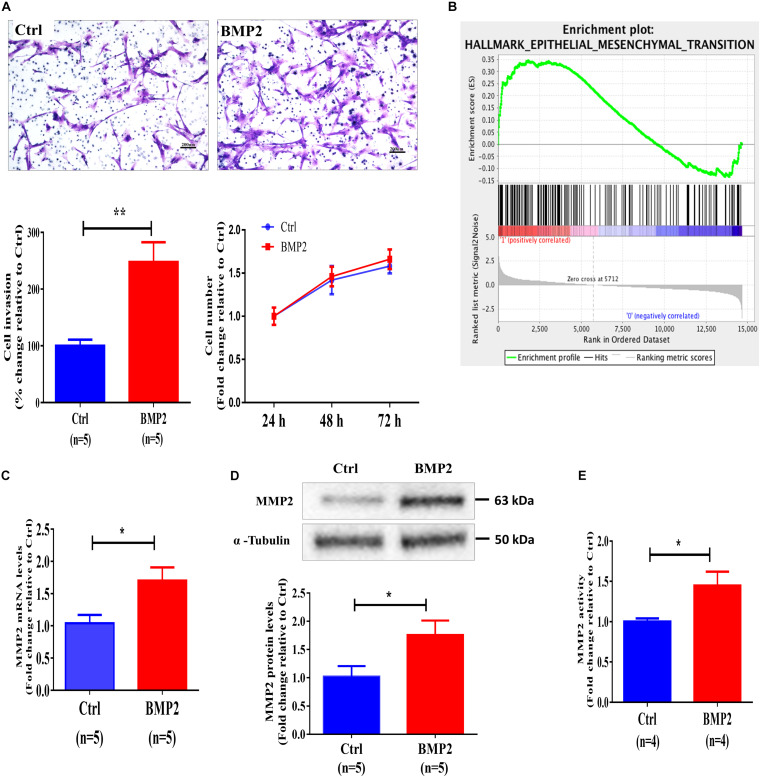
BMP2 upregulates MMP2 expression and promotes cell invasion in primary human EVT cells. **(A)** Primary human EVT cells were treated with a vehicle control (Ctrl) or 25 ng/mL BMP2 for 72 h, and cell invasion was examined using the Matrigel-coated transwell invasion assay. A representative image of the assay (scale bar represents 200 μm) and quantitative results are shown separately. Primary human EVT cells were seeded in 24-well plates, and the cells were treated every 24 h (24, 48 and 72 h) with Ctrl or 25 ng/mL BMP2 for a total of 72 h. Cell viability was determined using a CCK-8 assay. **(B)** The correlation between BMP2 treatment and the EMT signaling pathway sing the gene set enrichment analysis plot. **(C,D)** Primary human EVT cells were treated for 12 h **(C)** or 24 h **(D)** with Ctrl or 25 ng/mL BMP2 and the mRNA **(C)** and protein **(D)** levels of MMP2 were examined using RT-qPCR and western blot analyses, respectively. GAPDH and α-tubulin were used to normalize the RT-qPCR and western blot results, respectively. **(E)** Primary human EVT cells were treated for 24 h with Ctrl or 25 ng/mL BMP2 and MMP2 activity was examined using ELISA. The results are expressed as the mean ± SEM of five independent experiments (*n* = 5, *P < 0.05, **P < 0.01). Differences between groups were determined by Student’s *t*-test.

We further used primary human EVT cells isolated from first-trimester human placental villous tissues (normal pregnancies in women who were undergoing elective surgical termination at the gestational age of 5–8 weeks) to investigate the cellular activity of BMP2. To examine the bioactivity of exogenous BMP2, we treated primary EVT cells with 25 ng/mL BMP2 for 12 h, and the results showed that BMP2 significantly increased the mRNA levels of the inhibitor of differentiation (Id) proteins, ID1, ID2, and ID3 (Supplementary Figure 3). Using the Matrigel-coated transwell invasion assay and CCK-8 assay, we found that treatment of primary human EVT cells for 72 h with 25 ng/mL recombinant human BMP2 (BMP2) significantly increased cell invasion without affecting cell viability, indicating that BMP2 promotes cell invasion in primary human EVT cells (Figure 2A). Furthermore, the Gene Set Enrichment Analysis obtained from the gene sequencing results revealed that a high expression level of BMP2 was correlated with the EMT-related signaling pathway (Figure 2B). Matrix metalloproteases (MMPs), especially MMP2 and MMP9 are two gelatinases that are expressed in EVT cells and are associated with EMT-mediated trophoblast invasion during first-trimester pregnancy (Shimonovitz et al., 1994; Isaka et al., 2003). To investigate the effect of BMP2 on the expression of MMP2 and MMP9, we treated primary EVT cells with 25 ng/mL BMP2 for 12 or 24 h. The results showed that BMP2 significantly increased the mRNA and protein levels of MMP2 in primary EVT cells (Figures 2C,D). Moreover, treatment of primary EVT cells with 25 ng/mL BMP2 for 24 h increased MMP2 activity in the conditioned medium of cultured cells (Figure 2E). However, BMP2 did not have such effects on the expression of MMP9 and other MMPs (Supplementary Figure 4). These results indicate that BMP2 promotes cell invasion, most likely, by upregulating the expression of MMP2 in primary EVT cells.

### SNAIL Mediates the BMP2-Induced Upregulation of MMP2 and Increase in Cell Invasion in Primary Human EVT Cells

**FIGURE 3:**
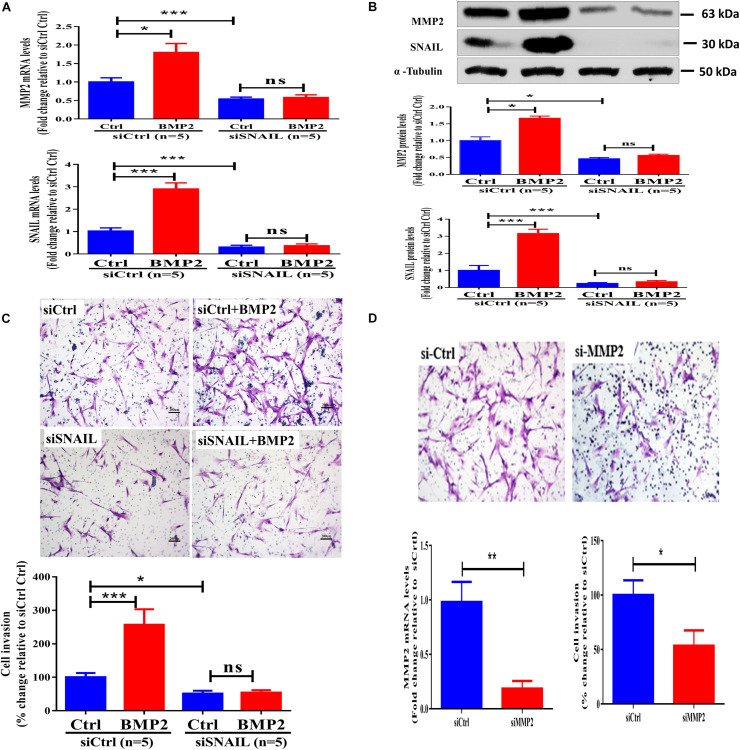
SNAIL mediates the BMP2-induced upregulation of MMP2 and the increase in cell invasion of primary human EVT cells. **(A,B)** Primary human EVT cells were transfected with 25 nM control siRNA (siCtrl) or 25 nM SNAIL-specific siRNA (siSNAIL) for 48 h, and the cells were treated with Ctrl or 25 ng/mL BMP2 for an additional 12 h **(A)** or 24 h **(B)**. The mRNA **(A)** and protein **(B)** levels of MMP2 and SNAIL were examined using the RT-qPCR and western blot results, respectively. **(C)** Primary human EVT cells were transfected with 25 nM siCtrl or 25 nM siSNAIL for 24 h, and the cells were treated with Ctrl or 25 ng/mL BMP2 for an additional 48 h. Cell invasion was examined using the Matrigel-coated transwell invasion assay. **(D)** Primary human EVT cells were transfected with 25 nM siCtrl or 25 nM siMMP2 for 24 h. The mRNA levels of MMP2 and cell invasion was examined using RT-qPCR and Matrigel-coated transwell invasion assay, respectively. The results are expressed as the mean ± SEM of five independent experiments (*n* = 5, ^∗^*P* < 0.05; ^∗∗∗^*P* < 0.001; NS, no significant difference). Differences between groups were determined by one-way analysis of variance.

The SNAIL protein is a prototypical EMT-inducing transcription factor that has been shown to mediate activin A-induced upregulation of MMP2 (Li et al., 2015). We thus investigated whether SNAIL plays a regulatory role in the BMP2-induced upregulation of MMP2 and increase in cell invasion. Using a siRNA-mediated knockdown approach, we found that the targeted depletion of SNAIL downregulated the mRNA and protein levels up to 80–90% (Figures 3A,B). Notably, SNAIL knockdown completely reversed the BMP2-induced upregulation of MMP2 expression (Figures 3A,B). Similarly, SNAIL knockdown completely abolished BMP2-induced increases in cell invasion (Figure 3C and Supplementary Figure 5). These results indicate that SNAIL is the main transcription factor that mediates the BMP2-induced trophoblast cell invasion. To further investigate the functional role of MMP2 in trophoblast invasion, we used siRNA-mediated knockdown approaches. The results showed that knockdown of MMP2 significantly decreased cell invasion in primary EVT cells (Figure 3D).

### MiR-502-5p Suppresses the Expression of SNAIL and Decreases SNAIL-Mediated Cell Invasion in Primary Human EVT Cells

**FIGURE 4:**
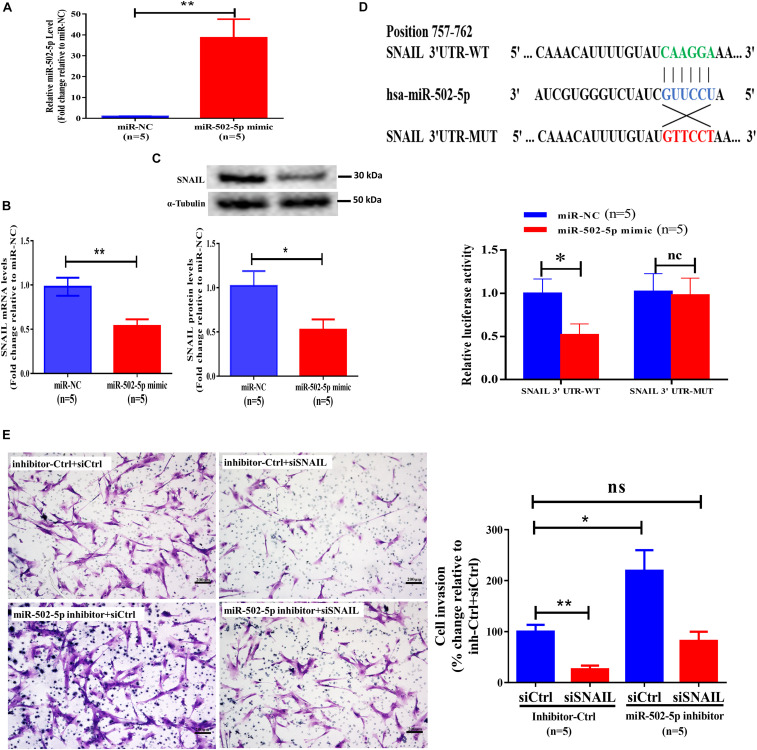
MiR-502-5p suppresses SNAIL expression and decreases SNAIL-mediated cell invasion in primary human EVT cells. **(A–C)** Primary human EVT cells were transfected for 24 h with 25 nM miR-NC (as a negative control) or 25 nM miR-502-5p mimic, and the miR-505-5p levels **(A)** were examined using RT-qPCR. The mRNA **(B)** and protein **(C)** levels of SNAIL were examined using RT-qPCR and western blot analyses, respectively. **(D)** Primary human EVT cells were transfected for 24 h with 3&2032;-UTR SNAIL-luciferase reporter (SNAIL 3&2032;-UTR-WT) or 3&2032;-UTR SNAIL mutant-luciferase reporter (SNAIL 3&2032;-UTR-MUT) along with 25 nM miR-NC or 25 nM miR-502-5p mimic. The luciferase activities of cells were detected using a dual-luciferase assay. **(E)** Primary human EVT cells were cotransfected with 25 nM miRNA inhibitor negative control (inhibitor Ctrl) or 25 nM miR-502-5p inhibitor for 24 h as well as 25 nM siCtrl or 25 nM siSNAIL for 24 h. Cell invasion was examined using the Matrigel-coated transwell invasion assay. The results are expressed as the mean ± SEM of five independent experiments (*n* = 5, *P < 0.05; **P < 0.001; NS, no significant difference). Differences between groups were determined by Student’s *t*-test or one-way analysis of variance.

To investigate the molecular mechanisms by which BMP2 regulates the expression of SNAIL, we performed bioinformatics analysis using TargetScan^1^. The results showed that the 3′ UTR of SNAIL mRNA contains a putative binding site of miR-502-5p. We thus treated primary EVT cells with 25 nM miR-NC (as a negative control) or 25 nM miR-502-5p mimic for 24 h, and the transfection efficiency was examined using RT-qPCR (Figure 4A). The results showed that transfection with miR-502-5p mimic significantly increased miR-502-5p level up to 40 folds (Figure 4A). Notably, the results showed that the miR-502-5p mimic significantly decreased the mRNA and protein levels of SNAIL (Figures 4B,C). To further confirm that SNAIL is the target of miR-502-5p, we cloned the SNAIL 3′ UTR sequence into a luciferase reporter construct (designated SNAIL 3′-UTR-WT) and mutated the putative miR-502-5p binding site (designated SNAIL 3′-UTR-MUT) (Figure 4D). Primary human EVT cells were transfected for 24 h with SNAIL 3′-UTR-WT or SNAIL 3′-UTR-MUT along with 25 nM miR-NC or 25 nM miR-502-5p mimic, and the luciferase activities of the cells were detected using a dual-luciferase assay. As shown in Figure 4D, compared with miR-NC, the miR-502-5p mimic significantly decreased the luciferase activity of SNAIL 3′-UTR-WT. However, the miR-502-5p mimic had no significant influence on the luciferase activity of the SNAIL 3′-UTR-MUT. Notably, the inhibition of miR-502-5p using the miR-502-5p inhibitor abolished the suppressive effect induced by the SNAIL knockdown (Figure 4E and Supplementary Figure 6). These results indicate that SNAIL is a direct target of miR-502-5p in primary EVT cells.

### ALK2 or ALK3 Mediates the BMP2-Induced Upregulation of SNAIL and MMP2 Expression in Primary Human EVT Cells

**FIGURE 5:**
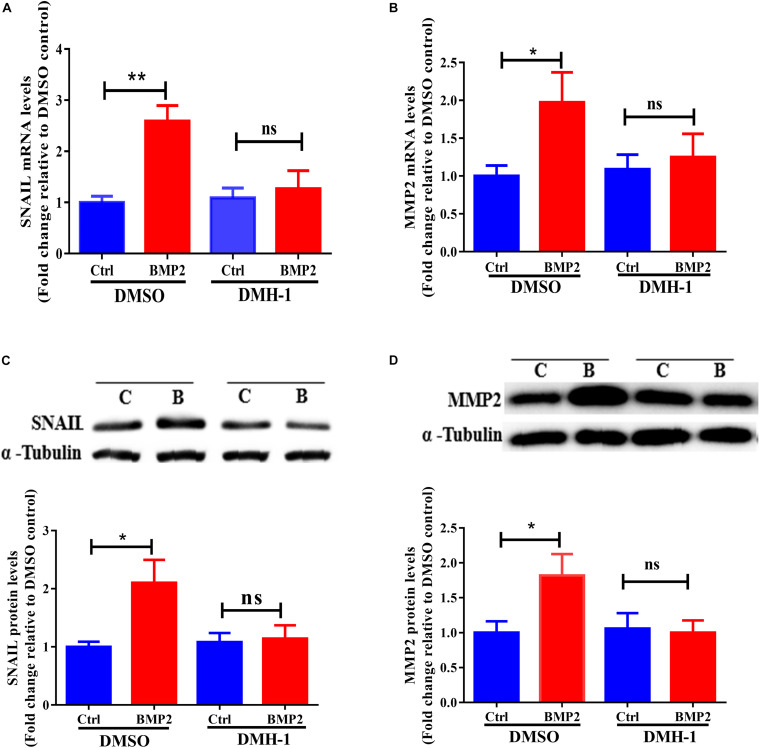
ALK2 or ALK3 mediates BMP2-induced upregulation of SNAIL and MMP2 in primary EVT cells. **(A,B)** Primary human EVT cells were pretreated with vehicle control or DMH-1 (0.5 μM) for 60 min and the cells were treated with Ctrl or 25 ng/mL BMP2 for an additional 12 h. The mRNA levels of SNAIL **(A)** and MMP2 **(B)** were examined using RT-qPCR. **(C,D)**, Primary human EVT cells were pretreated with vehicle control or DMH-1 (0.5 μM) for 60 min and the cells were treated with Ctrl or 25 ng/mL BMP2 for an additional 24 h. The protein levels of SNAIL **(C)** and MMP2 **(D)** were examined using western blot analysis. The results are expressed as the mean ± SEM of four independent experiments (*n* = 4, *P < 0.05; NS, no significant difference). Differences between groups were determined by one-way analysis of variance.

To date, three type I receptors (ALK2, ALK3, and ALK6) have been implicated in BMP-induced SMAD1/5/8 phosphorylation (Miyazono et al., 2001). To determine whether ALK2, ALK3 or ALK6 are required for BMP15-induced SMAD1/5/8 activation, primary EVT cells were treated with BMP2 in the presence or absence of 0.5 μM DMH-1 (a selective inhibitor of ALK2/3) (Yu et al., 2008). As shown in Figures 5A,B, treatment of primary EVT cells with DMH-1 completely abolished BMP2-induced increases in the mRNA levels of SNAIL and MMP2. Similarly, treatment of primary EVT cells with DMH-1 completely abolished BMP2-induced increases in the protein levels of SNAIL and MMP2 (Figures 5C,D). These results indicate that ALK2 or ALK3, but not ALK6, is required for the upregulation of SNAIL and MMP2 induced by BMP2 in primary EVT cells.

### NR026833.1 Induces SNAIL Expression and Promotes Cell Invasion in Primazry Human EVT Cells

**FIGURE 6:**
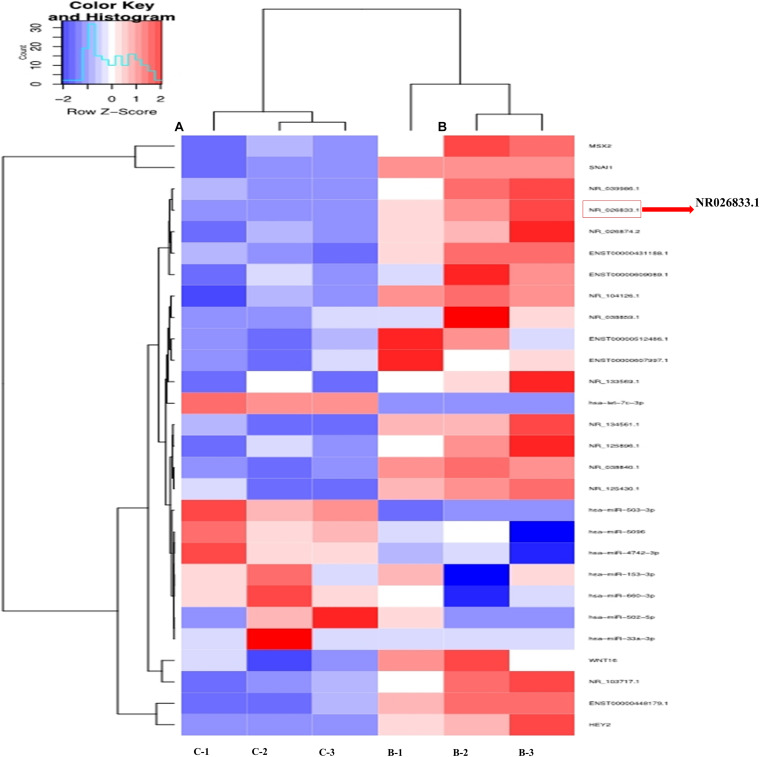
Hierarchical clustering analysis of lncRNA and microRNA expression in primary human EVT cells treated with Ctrl (*n* = 3) or 25 ng/mL BMP2 (*n* = 3). Each row represents one type of RNA, and each column represents a sample **(A)** Ctrl; **(B)** BMP2 treatment). The color scale shown at the top illustrates the relative RNA expression level; red represents high expression, and blue represents low expression.

**FIGURE 7:**
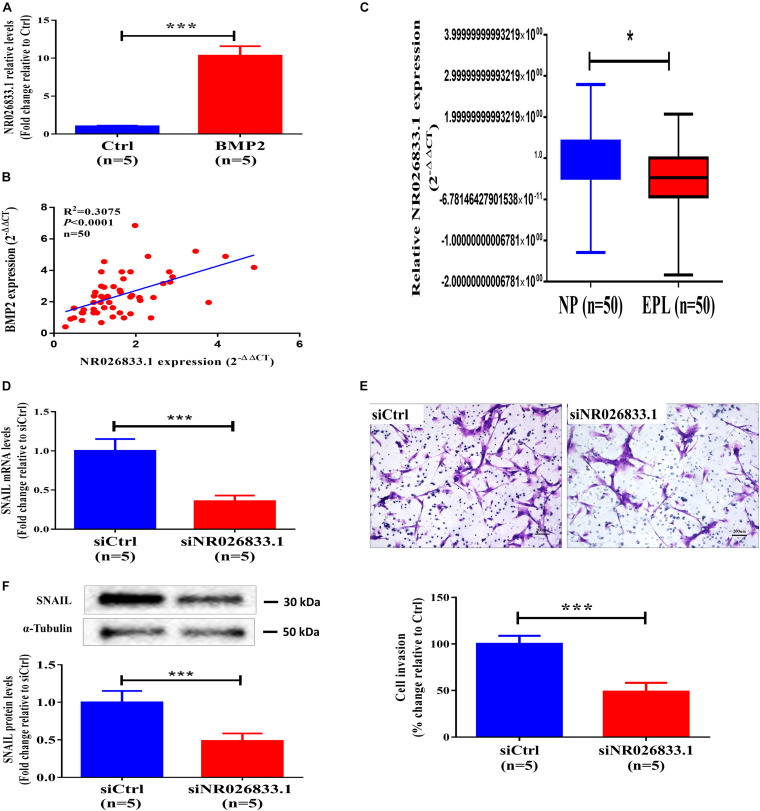
NR026833.1 induces SNAIL expression and promotes cell invasion in primary human EVT cells. **(A)** Primary human EVT cells were treated with Ctrl or 25 ng/mL BMP2 for 12 h, and the levels of NR026833.1 were examined using RT-qPCR. **(B)** Correlation of the relative mRNA levels of BMP2 and those of NR026833.1 in villous tissues obtained from women with NPs (*n* = 50). **(C)** The relative levels of NR026833.1 in villous tissue obtained from women with NPs (*n* = 50) and EPL (*n* = 50) were examined using RT-qPCR. **(D,E)** Primary human EVT cells were transfected with 25 nM siCtrl or 25 nM NR026833.1-specific siRNA (si NR026833.1) for 48 h, and the mRNA **(E)** and protein **(F)** levels of SNAIL were examined using RT-qPCR and western blot analyses, respectively. **(F)** Primary human EVT cells were transfected with 25 nM siCtrl or 25 nM si NR026833.1 for 48 h. Cell invasion was examined using a Matrigel-coated transwell invasion assay. The results are expressed as the mean ± SEM of at least five independent experiments (*P < 0.05; ***P < 0.001). Differences between groups were determined by Student’s *t*-test.

To further identify the regulatory networks of mRNAs, miRNAs, and lncRNAs, we treated primary human EVT cells (isolated from villous tissues obtained from three women with NPs) with 25 ng/mL BMP2 and performed high-throughput second-generation sequencing analysis. A total of 633 mRNAs (370 upregulated and 263 downregulated), 121 miRNAs (54 upregulated and 67 downregulated), and 908 lncRNAs (555 upregulated and 353 downregulated) were differentially expressed in the BMP2-treated group. Hierarchical clustering was performed to show the differential expression patterns of these mRNAs, miRNAs, and lncRNAs (Figure 6). Moreover, eight lncRNAs were selected for validation using RT-qPCR, and all these lncRNAs were upregulated by BMP2 according to the sequencing analysis (Supplementary Figure 7). Among these deregulated lncRNAs, NR026833.1 was upregulated up to 10-fold after BMP2 treatment (Figure 7A). Indeed, the results obtained from the correlation analysis showed that there was a positive correlation between the relative mRNA levels of BMP2 and those of NR026833.1 in villous tissues obtained from women with NPs (Figure 7B, *n* = 50). Intriguingly, the expression levels of NR026833.1 in the villous tissues obtained from women with NPs (*n* = 50) were significantly higher than those from women with EPL (Figure 7C, *n* = 50), indicating that NR026833.1 could play a functional role in early pregnancy. Our *in vitro* experiments showed that NR026833.1 knockdown significantly downregulated SNAIL expression at the transcriptional and translational levels (Figures 7D,E) and decreased cell invasion (Figure 7F) in primary EVT cells.

### NR026833.1 Binds to miR-502-5p and Promotes Cell Invasion in Primary Human EVT Cells

**FIGURE 8:**
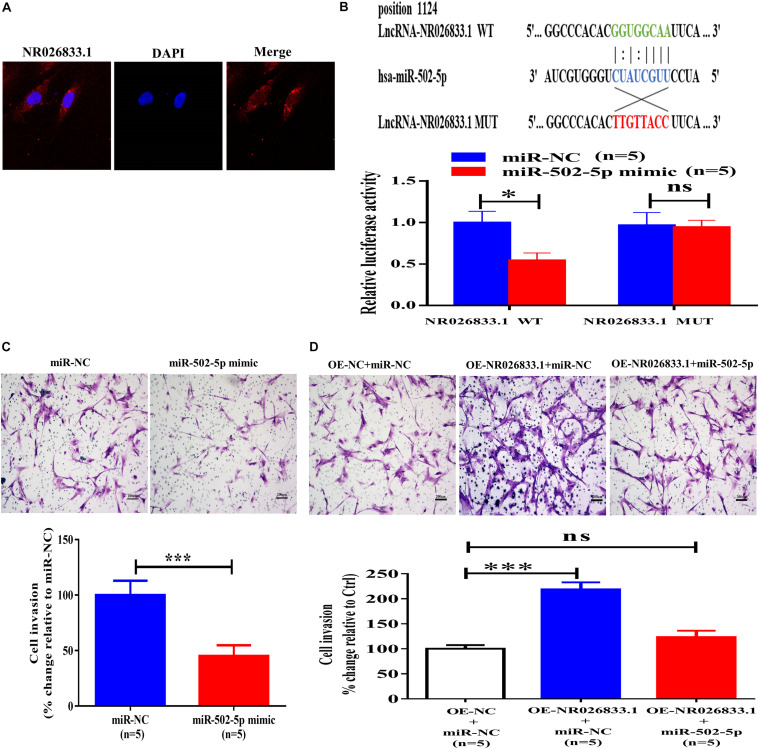
NR026833.1 binds to miR-502-5p and promotes cell invasion in primary human EVT cells. **(A)** Localization of NR026833.1 (red) in primary human EVT cells using immunostaining. DAPI staining (blue) was used to determine the location of the nuclei. **(B)** Primary human EVT cells were transfected for 24 h with an NR026833.1-luciferase reporter (NR026833.1-WT) or an NR026833.1 mutant-luciferase reporter (NR026833.1-MUT) along with 25 nM miRNA-NC or 25 nM miR-502-5p mimic. The luciferase activities of the cells were detected using a dual-luciferase assay. **(C)** Primary human EVT cells were transfected for 24 h with 25 nM miR-NC or 25 nM miR-502-5p mimic, and cell invasion was examined using a Matrigel-coated transwell invasion assay. **(D)** Primary human EVT cells were transfected for 24 h with miR-NC plus overexpression vector negative control (OE-NC), miR-NC plus overexpression NR026833.1 (OE-NR026833.1) or miR-502-5p mimic plus OE-NR026833.1, and cell invasion was examined using a Matrigel-coated transwell invasion assay. The results are expressed as the mean ± SEM of five independent experiments (*n* = 5, *P < 0.05; ***P < 0.001; NS, no significant difference). Differences between groups were determined by Student’s *t*-test.

The NR026833.1 gene is located at chromosome 2 and has 3,463 nucleotides. To investigate the molecular mechanisms by which NR026833.1 regulates trophoblast invasion, we examined the localization of this lncRNA because the cellular activities of lncRNAs are dependent on their subcellular distribution. Using cytoplasmic and nuclear RNA fractions combined with FISH analysis, we observed that NR026833.1 was preferentially localized in the cytoplasm of primary human EVT cells (Figure 8A). Studies have shown that cytoplasmic lncRNAs can directly bind to miRNAs and serve as microRNA sponges or competitive endogenous RNAs (ceRNAs) to block associations with target mRNAs (Zhou et al., 2014). Based on these studies and our sequencing results, we hypothesized that NR026833.1 has target sites against miR-502-5p and that NR026833.1 regulates trophoblast invasion by binding to miR-502-5p. To test this hypothesis, we constructed luciferase vectors by cloning wild-type NR026833.1 (NR026833.1 WT) and mutant NR026833.1 (NR026833.1 MUT). Using dual-luciferase assays, we found that transfection of the NR026833.1 WT together with the miR-502-5p mimic but not the NR026833.1 MUT with the miR-502-5p mimic significantly decreased the luciferase activity of primary EVT cells (Figure 8B). Additionally, transfection with the miR-502-5p mimic but not miR-NC significantly suppressed cell invasion in primary EVT cells (Figure 8C). Notably, overexpression of NR026833.1 (OE- NR026833.1) increased cell invasion and reversed the miR-502-5p mimic-induced suppression of cell invasion in primary EVT cells (Figure 8D). These results indicate that NR026833.1 promotes trophoblast invasion by interacting with miR-502-5p.

### BMP2 Promotes the Development of Mouse Preimplantation Embryos

**FIGURE 9:**
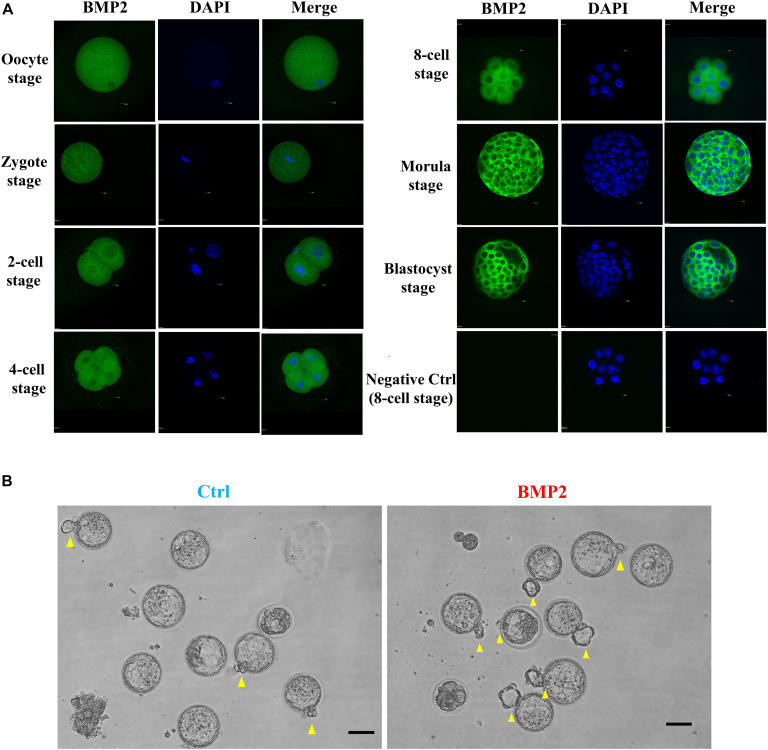
BMP2 promotes the development of mouse preimplantation embryos. **(A)** The expression and localization of BMP2 in mouse preimplantation embryos. Mouse preimplantation embryos, including the oocyte, zygote, 2-cell, 4-cell, 8-cell, morula and blastocyst stages, were fixed and probed with a specific antibody against mouse BMP2 followed by probing with a fluorescein isothiocyanate-conjugated secondary antibody or DAPI counterstaining. The images were detected using confocal microscopy. **(B)** BMP2 accelerates the processes of blastocyst formation and hatching. Mouse 2-cell embryos were cultured in HTF medium with Ctrl or 100 ng/mL BMP2 for 72 h. The processes of blastocyst formation and hatching (yellow arrowheads) were detected with microscopic observation. Six independent experiments (*n* = 6) were performed, and similar results were obtained (the total number of embryos in the control group was 110 and that in the BMP2 group was 111). The scale bars represent 50 μm.

To compare embryonic development in humans and mice, we further investigated the expression, localization and functional roles of BMP2 in mouse embryos at the corresponding stages. Using the fluorescence analysis, we observed that BMP2 is expressed in the mouse oocyte and preimplantation embryo at all stages, including the zygotes, 2-cell embryos, 4-cell embryos, 8-cell embryos, morula and blastocysts (Figure 9A). Specifically, BMP2 is primarily localized in the cytoplasm (Figure 9A). Similar to that in humans, BMP2 in mice is localized at the perinuclear area at the 2-cell stage. Additionally, BMP2 is expressed in both the trophectoderm and ICM (mainly in trophectoderm cells) at the blastocyst stage. To examine the roles of BMP2, we cultured superovulated 2-cell stage mouse embryos with 100 ng/mL BMP2 for up to 72 h. The results showed that BMP2 significantly promoted blastocyst formation at 48 h and embryo hatching at 72 h (Figure 9B and Table 2).

**TABLE 2:** Effects of BMP2 on the developmental potential of 2-cell mouse embryos.

		**48 h**	**72 h**	
		
		**No. of blastocyst (%)**	**No. of blastocyst (%)**	**No. of blastocyst hatching (%)**
Control	110^a^	51 (46.4)	95 (86.4)	53 (48.2)
BMP2	111^a^	74 (66.7)*	101 (91)	79 (71.2)*
*P-*value		0.002	0.278	0.002

^*a*^Number of embryos that were examined.*P < 0.05 compared with Control.

### BMP2 Promotes the Invasive Differentiation of Mouse TS Cells

**FIGURE 10:**
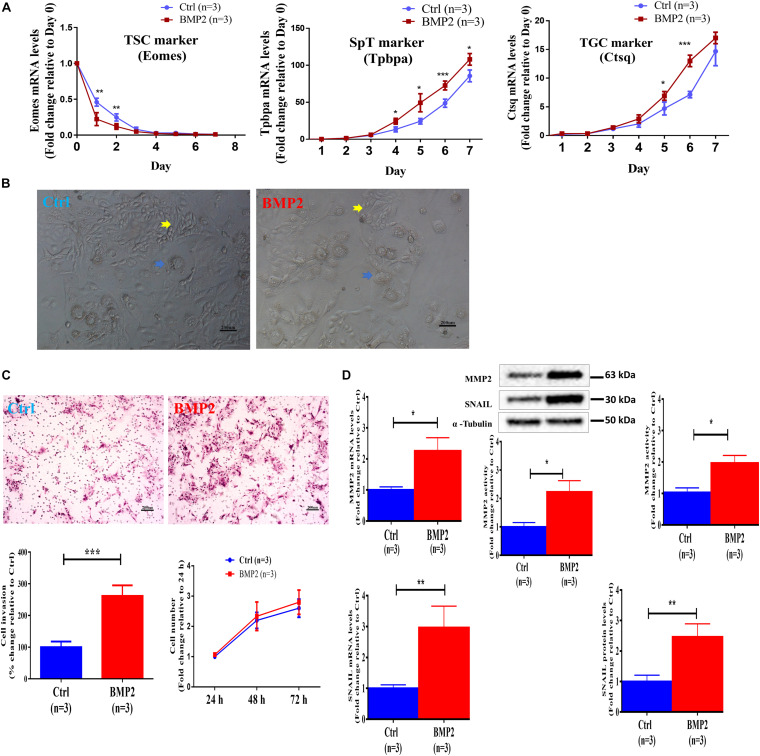
BMP2 promotes the invasive differentiation of mouse TS cells. **(A)** TS cells were treated with Ctrl or 100 ng/mL BMP2 every 24 h for up to 7 days, and three cell marker genes, including *Eomes* (TS cell marker), *Tpbpa* (SpT marker) and *Ctsq* (TGC marker), were examined using RT-qPCR. **(B)** TS cells were treated with Ctrl or 100 ng/mL BMP2 for 4 days, and cell images were detected with microscopic observation. Undifferentiated TS cells showed a colony with characteristic tightly packed cells (yellow arrowheads), while differentiated TS cells showed a typical TGC morphology (blue arrowheads). These differentiated TS cells showed a flattened appearance and increased cell, nuclei and perinuclear granule sizes (blue arrowheads). The scale bars represent 200 μm. **(C)** TS cells were treated with Ctrl or 100 ng/mL BMP2 every 24 h for a total of 72 h, and cell invasion was examined using a Matrigel-coated transwell invasion assay. Cell viability was determined using a CCK-8 assay. **(D)** TS cells were treated for 12 or 24 h with Ctrl or 100 ng/mL BMP2, and the mRNA (12 h) and protein (24 h) levels of MMP2 and SNAIL were examined using RT-qPCR and western blot analyses, respectively. TS cells were treated for 24 h with Ctrl or 100 ng/mL BMP2, and the MMP2 activity was examined using ELISA. GAPDH and α-tubulin were used to normalize the RT-qPCR and western blot results, respectively. The results are expressed as the mean ± SEM of three independent experiments (*n* = 3, *P < 0.05, **P < 0.01 and ***P < 0.001). Differences between groups were determined by Student’s *t*-test or one-way analysis of variance.

To investigate the role of BMP2 in the regulation of trophoblast cell differentiation and invasion, we used mouse TS cells (established from 3.5 days postcoitus (dpc) blastocysts) to mimic the process of trophoblast differentiation *in vivo*. Our *in vitro* studies showed that BMP2 treatment promptly decreased the expression of the TS cell marker Eomes during the first 3 days of cell differentiation (Figure 8A). BMP2 increased the expression of the labyrinthine/spongiotrophoblast (SpT) cell marker Tpbpa and the trophoblast giant cell (TGC) marker Ctsq during the last 3 days of cell differentiation (Figure 10A). Specifically, after BMP2 treatment, the number of undifferentiated TS cells (a colony with tightly packed cells characteristic, yellow arrowheads) was decreased, while the number of differentiated TS cells (a typical TGC morphology, blue arrowheads) (Figure 10B). These results indicated that recombinant BMP2 promotes TS cell differentiation. Similar to the results in humans, BMP2 promoted cell invasion without affecting cell proliferation in mouse stem cells (Figure 10C). Additionally, BMP2 increased the expression of SNAIL and MMP2 at the transcriptional and translational levels in mouse TS cells (Figure 10D). Moreover, BMP2 significantly increased the MMP2 activity in the conditioned medium of cultured cells (Figure 10D). These results indicate that BMP2 promotes the invasive differentiation of mouse trophoblasts.

## Discussion

Trophoblast differentiation and invasion are critical for early embryo development and subsequent implantation, and any dysregulation of the key regulators in this process may lead to an unsuccessful pregnancy. Members of the TGF-β superfamily are involved in the regulation of embryo implantation and trophoblast invasion (Cheng et al., 2013; Li et al., 2014). Additionally, studies have shown that members of the BMP subfamily induce differentiation of cells to the trophoblast lineage (Xu et al., 2002; Lichtner et al., 2013). Our previous study also showed that BMP2 promotes human trophoblast cell invasion by upregulating EMT-associated markers (Zhao et al., 2018a,b, 2020). In this follow-up study, we further investigated the function and molecular mechanisms by which BMP2 regulates the process of embryo development and trophoblast differentiation. Information obtained from clinical samples showed that the serum BMP2 levels were significantly increased during the first trimester of pregnancy. Given that elevated peripheral BMP2 levels are associated with the development of placental tissue during the first trimester, trophoblast cells are one of the sources of the circulating BMP2. Additionally, the serum BMP2 levels were significantly reduced and the BMP2 expression levels were downregulated in villous tissues obtained from women with EPL, indicating that BMP2 is a principal factor for placentation in humans. Moreover, we provided the first data showing that BMP2 is expressed in the human oocyte and trophoblast cells of cleavage embryos and blastocysts prior to implantation. However, all the human embryos used in this study were embryos developing slowly or with arrested development and those with poor-quality embryo morphology. These embryos were selected not to be used for embryo transfer during assisted reproductive technology. Therefore, the expression level of BMP2 in these embryos could be different from those in the normal developed embryos. Previous studies have shown that embryo implantation promotes endometrium decidualization (Paria et al., 2001; Sharma et al., 2016). Taken together, previous studies and our results indicate that BMP2 secreted by endometrial cells and trophoblast cells may mediate their cooperation during placentation in an autocrine/paracrine manner, which is essential for early pregnancy maintenance.

In this study, the gene set enrichment analysis plot indicated a significant correlation between BMP2 and the EMT signaling pathway. EMT is a fundamental process of cell shape change that forms extravillous and interstitial cytotrophoblasts with mesenchymal characteristics during placental development (Kokkinos et al., 2010). MMPs have been reported to be related to the invasive ability of trophoblasts, which are capable of digesting collagen IV, a major component of the basement membrane (Seval et al., 2004). A previous study showed that MMP2 and MMP9 were localized at the placental bed, primarily in EVT cells during early pregnancy, and were involved in the regulation of trophoblast invasion (Ma et al., 2015). Specifically, MMP2 is expressed at a peak level, which is closely associated with invasive potential at the implantation site during the first trimester of pregnancy (Shimonovitz et al., 1994; Isaka et al., 2003; Bai et al., 2005; Jones et al., 2006). Notably, we found that BMP2 promoted trophoblast invasion by upregulating expression of MMP2 but not expression of MMP9. In the trophoblast cells obtained from a gestational placenta at 6–8 weeks, MMP2 is a key regulator of trophoblast invasion (Staun-Ram et al., 2004). Consistent with these results, our *in vitro* experiments confirmed that BMP2 plays a key role in regulating trophoblast invasion by upregulating MMP2 expression through SNAIL.

In the present study, our functional studies revealed that BMP2 has a regulatory role in the promotion of trophoblast invasion in primary EVT cells. Using whole-genome sequencing, we identified certain differentially expressed mRNAs, miRNAs and lncRNAs that are associated with BMP2-induced trophoblast invasion. Many studies have demonstrated the involvement of miRNAs and lncRNAs in the regulation of trophoblast function and placental development in humans (McAninch et al., 2017; Hayder et al., 2018). LncRNAs are defined as RNAs longer than 200 nucleotides in length and do not produce a protein product. Here, we showed that BMP2 prominently induces the upregulation of NR026833.1, a lncRNA expressed in primary human EVT cells. Indeed, the expression levels of BMP2 were positively correlated with those in human villous tissue. Clinical data also showed that the expression levels of NR026833.1 were significantly reduced in villous tissues obtained from women with EPL compared to those obtained from women with NPs, indicating that this lncRNA has a role in early placentation. Our experiments further demonstrated that NR026833.1 knockdown decreased cell invasion, whereas NR026833.1 overexpression increased cell invasion in primary EVT cells. LncRNA can serve as a miRNA sponge to sequester and saturate the cellular pool of miRNA, thereby operating as a competitive inhibitor to suppress the binding of miRNA to its mRNA targets (Bak and Mikkelsen, 2014). We further confirmed that NR026833.1 was preferentially localized in the cytoplasm of primary human EVT cells and that there was a binding site in NR026833.1 for miR-502-5p, a miRNA that was also expressed in primary human EVT cells. Notably, the miR-502-5p mimic abolished the cell invasion-promoting effect caused by NR026833.1 overexpression. These results indicate that NR026833.1 may serve as an intracellular sponge for miR-502-5p and may abolish the miR-502-5p-induced suppression of cell invasion. MiRNAs are classified as endogenous small non-coding RNAs that negatively modulate gene expression by binding to specific mRNAs and promoting their degradation or translational repression (Bartel, 2004). Using bioinformatics analysis and luciferase reporter assays, we identified a putative binding site of the 3′ UTR of SNAIL mRNA that specifically matched miR-502-5p, indicating that SNAIL is the target of this trophoblast-derived miRNA. Our study further revealed that transfection with the miR-502-5p mimic suppressed the expression of SNAIL at the transcriptional and translational levels and that inhibition of miR-502-5p reversed the siSNAIL-mediated decrease in cell invasion. SNAIL is a prototypical EMT-inducing transcription factor that is essential for trophoblast invasion (Blechschmidt et al., 2007). Using the siRNA-mediated inhibition approach, we showed that SNAIL knockdown decreased basal and BMP2-induced cell invasion in primary EVT cells. These findings suggest that in human primary EVT cells, miR-502-5p acts to suppress cell invasion by binding to the EMT-related transcription factor SNAIL and that the BMP2-induced increase in NR026833.1 promotes the expression of SNAIL by competitively binding to (and suppressing the effect of) miR-502-5p.

Improper trophectoderm differentiation is associated with arrested preimplantation development and defective embryo implantation, leading to early pregnancy failure (Roberts and Fisher, 2011; Pfeffer and Pearton, 2012). Because of ethical issues, we utilized mouse embryos to investigate the effects of BMP2 on embryo development and trophoblast invasion. The results showed that BMP2 is expressed in the mouse oocyte and during all embryo stages, including 2, 4, and 8-cell embryos as well as morula and blastocysts (mainly in the trophectoderm cells). Intriguingly, exogenous BMP2 promoted embryonic development by enhancing blastocyst formation and hatching in mice. In line with our results, a previous study showed that several BMP2 receptor genes, including Bmpr1a, are expressed in the extraembryonic ectoderm (ExE) (Kishigami and Mishina, 2005). *In situ* hybridization studies in mice demonstrated that the expression of BMP2 in the uterus was spatiotemporally correlated with embryo implantation, suggesting that BMP2 plays a critical role during embryo implantation and early placentation (Paria et al., 2001). Additionally, we used mouse TS cells to examine the roles of BMP2 in the regulation of trophoblast cell differentiation and invasion. Our results showed that treatment with BMP2 promoted trophoblast differentiation by downregulating the expression of Eomes (TS cell marker) and upregulating the expression of Ctsq (TGC marker) and Tpbpa (SpT marker) in mouse TS cells. Additionally, exogenous BMP2 promoted cell invasion without affecting cell proliferation. Similar to the results in primary human EVT cells, the BMP2-induced increase in cell invasion is most likely through the upregulation of SNAIL and MMP2 expression in mouse TS cells (Figure 9). These findings suggest that the stimulatory effect of BMP2 on the invasive potential of trophoblasts most likely occurs at a very early stage of placentation. However, one question has been raised regarding the cause and effect of BMP2 and EPL in humans. Indeed, it is difficult to draw a conclusion whether changes in the levels of BMP2 are a cause or effect of the pregnancy loss in humans. Future studies aimed at addressing this issue will be of great interest.

## Conclusion

**FIGURE 11:**
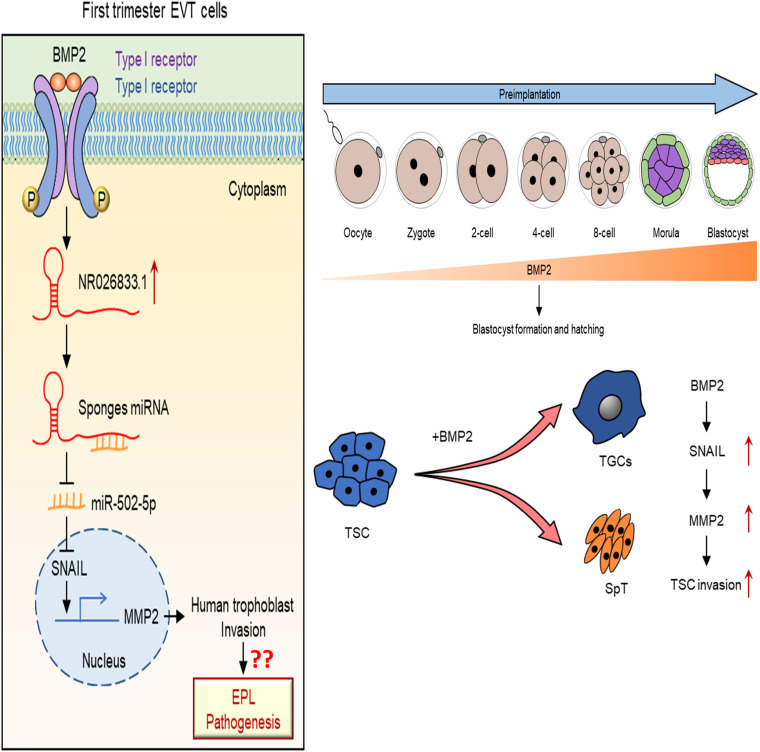
A **s**chematic diagram of the regulatory role of the BMP2/NR026833.1/SNAIL/MMP2 signaling axis in promoting the invasive differentiation of trophoblasts. Left panel figure. LncRNA NR026833.1 is upregulated by BMP2 and promotes the expression of SNAIL (target gene of miR-502-5p) by acting as a decoy to competitively bind to miR-502-5p. The upregulation of SNAIL further acts as a transcription factor that induces the production of MMP2, which in turn promotes cell invasion in primary EVT cells. Right panel figure. In humans and mice, BMP2 is expressed in the oocyte and all embryo stages (especially the trophectoderm). Exogenous BMP2 enhances embryonic development by increasing blastocyst formation and hatching. Additionally, BMP2 promotes the invasive differentiation of TS cells by upregulating the expression of SNAIL and MMP2.

In conclusion, our findings provide significant insights into the molecular biology of embryo-maternal interactions, underscoring the importance of BMP2 in promoting blastocyst implantation and placental development. These processes are essential for successful pregnancy and fetal development and growth. Notably, our study reveals a new regulatory pathway in which BMP2 induces a miR502-5p/SNAIL/MMP2 signaling axis via NR026833.1 upregulation (Figure 11). This advance in knowledge will provide us a framework to explore new diagnostic and therapeutic strategies for pregnancy-related complications.

## Data Availability Statement

The original contributions presented in the study are included in the article/Supplementary Material, further inquiries can be directed to the corresponding author/s.

## Ethics Statement

The studies involving human participants were reviewed and approved by the Ethical Review Committee of Women’s Hospital, Zhejiang University School of Medicine. The patients/participants provided their written informed consent to participate in this study. The animal study was reviewed and approved by the Ethical Review Committee of Women’s Hospital, Zhejiang University School of Medicine.

## Author Contributions

Y-MZ and PL designed the research. JY and WW performed the research and analyzed the data. JY and H-MC wrote the manuscript. JY, HZha, HZhu, YS, and MT collected first trimester human placental villi. CW, YS, GF, and SC analyzed and interpreted the data. Y-MZ and PL revised the manuscript. All authors were involved in interpreting the data and approved the final article.

## Conflict of Interest

The authors declare that the research was conducted in the absence of any commercial or financial relationships that could be construed as a potential conflict of interest.

## Footnotes

1 http://www.targetscan.org/vert_50/

## Supplementary Material

Supplementary Figure 1 The purity of EVT cell cultures was verified by immunocytochemical staining for cytokeratin-7. DAPI was used to stain the cell nuclei.

Supplementary Figure 2 A whole image of western blot showing the location of BMP2 (molecular weight: 44 kDa).

Supplementary Figure 3 BMP2 upregulates the expression of ID1, ID2, and ID3 in primary human EVT cells. Primary human EVT cells were treated for 12 h with Ctrl or 25 ng/mL BMP2 and the mRNA levels of ID1, ID2, and ID3 were examined using RT-qPCR. GAPDH was used to normalize the RT-qPCR results. The results are expressed as the mean ± SEM of five independent experiments (*n* = 5, ^∗∗∗^*P* < 0.001). Differences between groups were determined by Student’s *t*-test.

Supplementary Figure 4 Primary human EVT cells were treated for 12 h with Ctrl or 25 ng/mL BMP2 and the mRNA levels of MMP9 **(A)** and MMP1/3/7/10/11/12/14/15/16/17/21/23/24/25/28 **(B)** were examined using RT-qPCR. The results are expressed as the mean ± SEM of five independent experiments (*n* = 5, NS, no significant difference). Differences between groups were determined by Student’s *t*-test.

Supplementary Figure 5 SNAIL mediates the BMP2-induced upregulation of MMP2 and the increase in cell invasion of primary human EVT cells. Primary human EVT cells were transfected with 25 nM siCtrl or 25 nM siSNAIL for 24 h, and the cells were treated with Ctrl or 25 ng/mL BMP2 for an additional 48 h. Cell invasion was examined using the Matrigel-coated transwell invasion assay.

Supplementary Figure 6 MiR-502-5p suppresses SNAIL expression and decreases SNAIL-mediated cell invasion in primary human EVT cells. Primary human EVT cells were cotransfected with 25 nM miRNA inhibitor negative control (inhibitor Ctrl) or 25 nM miR-502-5p inhibitor for 24 h as well as 25 nM siCtrl or 25 nM siSNAIL for 24 h. Cell invasion was examined using the Matrigel-coated transwell invasion assay.

Supplementary Figure 7 **(A,B)** Volcano plot of the *P*-values as a function of weighted fold-change for lncRNAs in primary human EVT cells treated with BMP2. Gray dots represent lncRNAs not significantly differentially expressed (fold change < 1; *P* > 0.05) and red and green dots represent lncRNAs that are significantly differentially expressed (fold change ≥ 1; *P* < 0.05). **(C)** Primary human EVT cells were treated for 24 h with Ctrl or 25 ng/mL BMP2 and the relative levels of various lncRNA were examined using RT-qPCR.

Supplementary Data 1 The list of primers sequence.

Supplementary Data 2 The list of antibodies used in this study.
