# Supplementary material for: The BMP2 Signaling Axis Promotes Invasive Differentiation of Human Trophoblasts
Source: Front Cell Dev Biol. 2021 Feb 4;9:607332. doi: 10.3389/fcell.2021.607332 (PMC7889606; doi:10.3389/fcell.2021.607332)
Supplement: Supplementary Figure 1 — The purity of EVT cell cultures was verified by immunocytochemical staining for cytokeratin-7. DAPI was used to stain the cell nuclei. [file Data_Sheet_3.DOC]

**Supplemental Figure Legends:**

**Supplemental Figure 1**


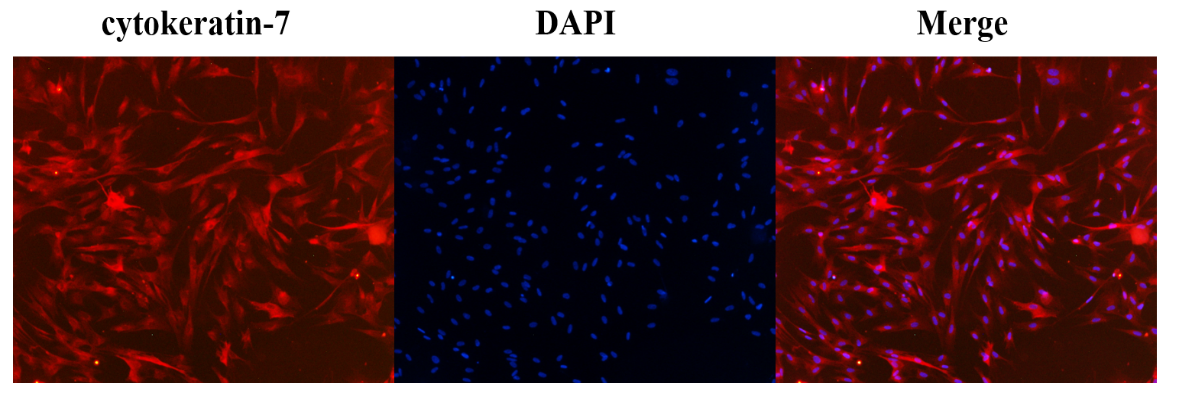


**Supplemental Figure 1.** The purity of EVT cell cultures was verified by immunocytochemical staining for cytokeratin-7. DAPI was used to stain the cell nuclei.

**Supplemental Figure 2**

**
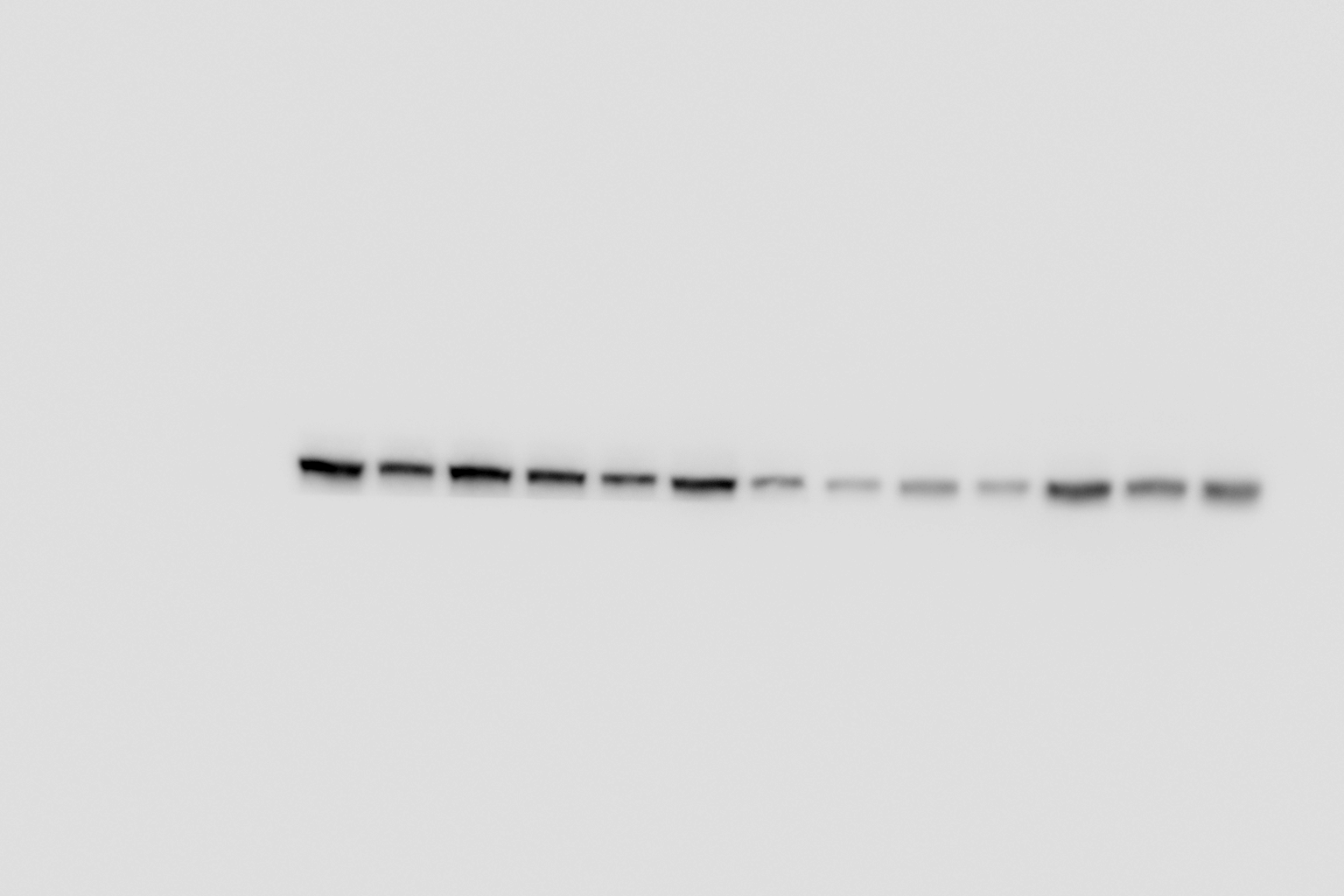
**

**Supplemental Figure 2.** A whole image of western blot showing the location of BMP2 (molecular weight: 44 kDa).

**Supplemental Figure 3**

**Supplemental Figure 3.** BMP2 upregulates the expression of ID1, ID2 and ID3 in primary human EVT cells. Primary human EVT cells were treated for 12 h with Ctrl or 25 ng/mL BMP2 and the mRNA levels of ID1, ID2 and ID3 were examined using RT-qPCR. GAPDH and α-tubulin were used to normalize the RT-qPCR and western blot results, respectively. The results are expressed as the mean ± SEM of five independent experiments (n=5, ***P < 0.001). Differences between groups were determined by Student’s t-test.

**Supplemental Figure 4**

**A**

**NS**

**B**


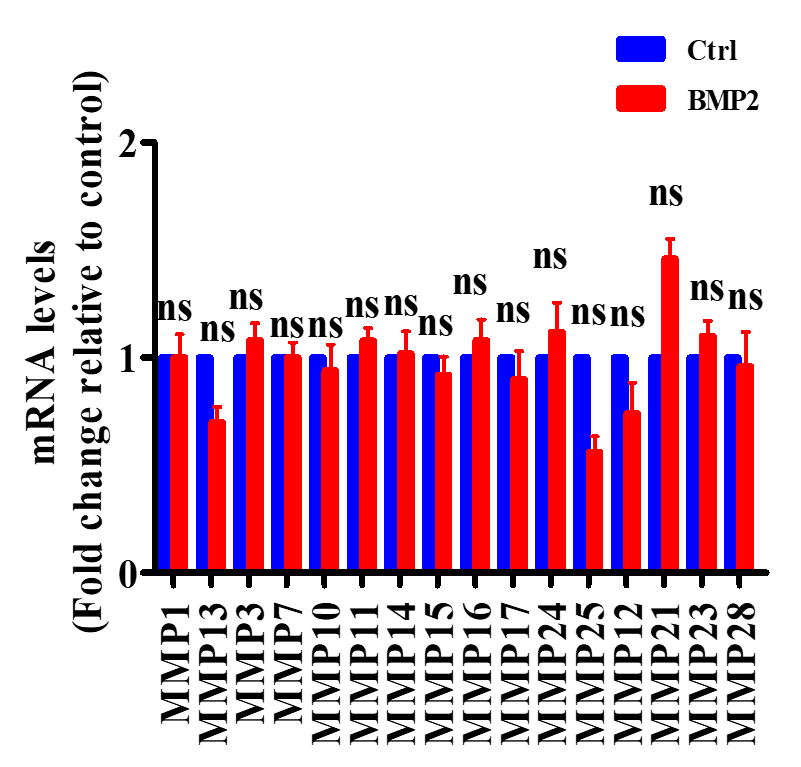


**Supplemental Figure 4.** Primary human EVT cells were treated for 12 h with Ctrl or 25 ng/mL BMP2 and the mRNA levels of MMP9 (A) and MMP1/3/7/10/11/12/14/15/16/17/21/23/24/25/28 (B) were examined using RT-qPCR. The results are expressed as the mean ± SEM of five independent experiments (n=5, NS, no significant difference). Differences between groups were determined by Student’s t-test.

**Supplemental Figure 5**


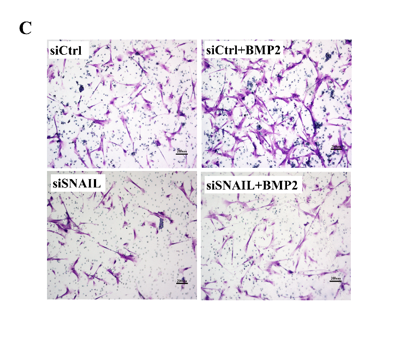


**Supplemental Figure 5. SNAIL mediates the BMP2-induced upregulation of MMP2 and the increase in cell invasion of primary human EVT cells.** Primary human EVT cells were transfected with 25 nM siCtrl or 25 nM siSNAIL for 24 h, and the cells were treated with Ctrl or 25 ng/mL BMP2 for an additional 48 h. Cell invasion was examined using the Matrigel-coated transwell invasion assay.

**Supplemental Figure 6**


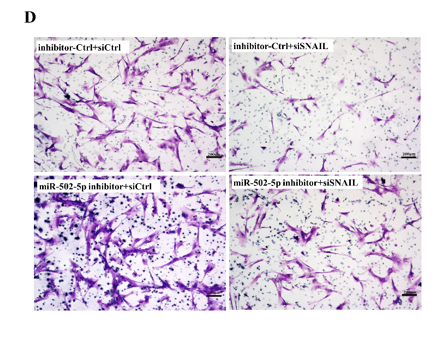


**Supplemental Figure 6. MiR-502-5p suppresses SNAIL expression and decreases SNAIL-mediated cell invasion in primary human EVT cells.** Primary human EVT cells were cotransfected with 25 nM miRNA inhibitor negative control (inhibitor Ctrl) or 25 nM miR-502-5p inhibitor for 24 h as well as 25 nM siCtrl or 25 nM siSNAIL for 24 h. Cell invasion was examined using the Matrigel-coated transwell invasion assay.

**Supplemental Figure 7**

**
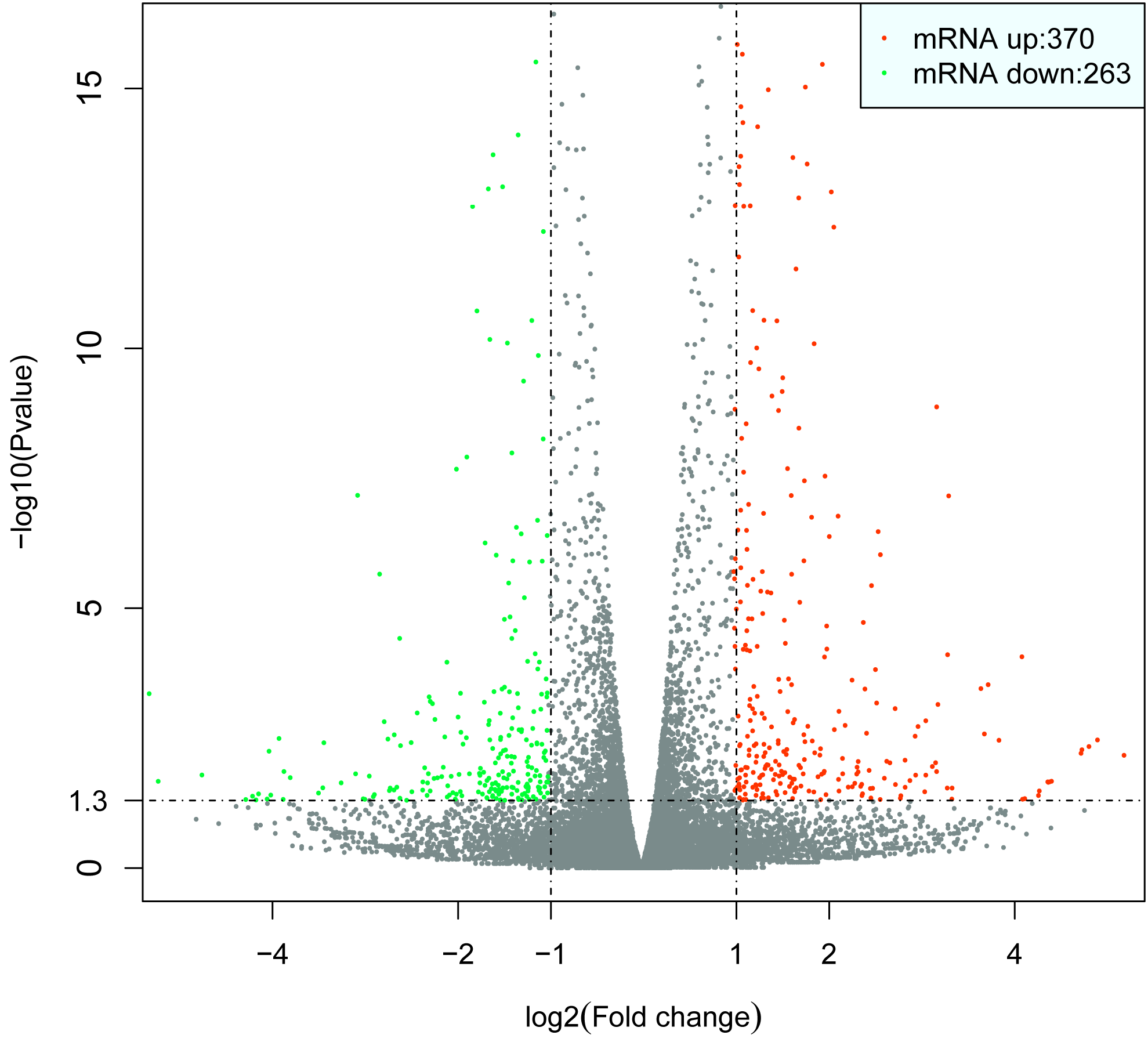
Supplemental Figure 7.** A and B, Volcano plot of the P-values as a function of weighted fold-change for lncRNAs in primary human EVT cells treated with BMP2. Grey dots represent lncRNAs not significantly differentially expressed (fold change <1; P>0.05) and red and green dots represent lncRNAs that are significantly differentially expressed (fold change ≥1; P<0.05). C, Primary human EVT cells were treated for 24 h with Ctrl or 25 ng/mL BMP2 and the relative levels of various lncRNA were examined using RT-qPCR.

**LncRNAs**

**mRNA**

**B**

**C**
