# Supplementary material for: The BMP2 Signaling Axis Promotes Invasive Differentiation of Human Trophoblasts
Source: Front Cell Dev Biol. 2021 Feb 4;9:607332. doi: 10.3389/fcell.2021.607332 (PMC7889606; doi:10.3389/fcell.2021.607332)
Supplement: Supplementary Data 1 — The list of primers sequence. [file Data_Sheet_1.DOC]

**Supplemental Data 1.** The list of primers sequence

| Gene | Primer sequence |
| --- | --- |
| Loop R primer | CAGTGCAGGGTCCGAGGTAT |
| Human-GAPDH-F | GAGTCAACGGATTTGGTCGT |
| Human-GAPDH-R | GACAAGCTTCCCGTTCTCAG |
| Human-BMP2-F | CTCCACTAATCATGCCATTGTTCAGAC |
| Human-BMP2-R | CGTCAAGGTACAGCATCGAGATAGC |
| Human-NR026833.1-F | GAATATCAGGCACACGGCCA |
| Human-NR026833.1-R | GACACAGATGCAGTTTGGCG |
| Human-SNAIL-F | CCCCAATCGGAAGCCTAACT |
| Human-SNAIL-R | GCTGGAAGGTAAACTCTGGATTAGA |
| Human-MMP2-F | TACACCAAGAACTTCCGTCTGT |
| Human-MMP2-R | AATGTCAGGAGAGGCCCCATA |
| Human-MMP9-F | GGACGATGCCTGCAACGT |
| Human-MMP9-R | CAAATACAGCTGGTTCCCAATCT |
| mouse-GAPDH-F | AAGCCCATCACCATCTTCCA |
| mouse-GAPDH-R | CCTGCCTCACCACCTTCTTG |
| mouse-Eomes-F | CACTGGATGAGGCAGGAGAT |
| mouse-Eomes-R | CGGTTGGTATTTGTGCAGAG |
| mouse-Ctsq-F | CAAGGCAATAGAGGCTGTCG |
| mouse-Ctsq-R | TTCACTTTCTGGGAGGACCA |
| mouse-Tpbpa-F | CCAGTTGTTGATGACCCTGA |
| mouse-Tpbpa-R | CTGTTTCGCTCGTTGCCTA |
| mouse-MMP2-F | CACCACCGAGGACTATGACC |
| mouse-MMP2-R | CCAGGAAAGTGAAGGGGAAG |
| mouse-SNAIL-F | CCATTCTCCTGCTCCCACT |
| mouse-SNAIL-R | CCTGGCACTGGTATCTCTTCA |
