# Supplementary material for: The BMP2 Signaling Axis Promotes Invasive Differentiation of Human Trophoblasts
Source: Front Cell Dev Biol. 2021 Feb 4;9:607332. doi: 10.3389/fcell.2021.607332 (PMC7889606; doi:10.3389/fcell.2021.607332)
Supplement: Supplementary Data 2 — The list of antibodies used in this study. [file Data_Sheet_2.DOC]

**Supplemental data S2. The list of antibodies used in this study.**

| Protein | Cat. No. | Company |
| --- | --- | --- |
| α-tubulin | B-5-1-2; sc-23948 | Santa Cruz Biotechnology |
| BMP2 | ab6285 | Abcam |
| SNAIL | no. 3895 | Cell Signaling Technology |
| MMP2 | ab86607 | Abcam |
| Cytokeratin-7 | MAB3554 | EMD Millipore |
| Rabbit IgG1 isotype | ab172730 | Abcam |
