# Supplementary material for: The BMP2 Signaling Axis Promotes Invasive Differentiation of Human Trophoblasts
Source: Front Cell Dev Biol. 2021 Feb 4;9:607332. doi: 10.3389/fcell.2021.607332 (PMC7889606; doi:10.3389/fcell.2021.607332)
Supplement: Supplementary file 4 [file Table_1.DOCX]

**Table 1.** **Clinical and biochemical characteristics of the women included in this study.**

|  | Non-pregnacy (Non-P, n=50) | Normal pregnancy (NP, n=50) | Early pregnancy loss  (EPL, n=50) |
| --- | --- | --- | --- |
| Age (year) | 28.04±3.14 | 28.06±4.65 | 28.92±3.48 |
| Gestational age (day) | - | 47.06±4.99 | 48.76±4.18 |
| Serum β-HCG (IU/mL) | - | 38,427.18±41,159.79^a^ | 22,510.28±30,457.91^a^ |
| Serum BMP2 (pg/mL) | 36.28±13.82^b^ | 51.02±18.77^bc^ | 43.16±16.35^c^ |

Data are presented as the means ± SD. a, P<0.05; b, P<0.001; c, P<0.05
