# Supplementary material for: The BMP2 Signaling Axis Promotes Invasive Differentiation of Human Trophoblasts
Source: Front Cell Dev Biol. 2021 Feb 4;9:607332. doi: 10.3389/fcell.2021.607332 (PMC7889606; doi:10.3389/fcell.2021.607332)
Supplement: Supplementary file 5 [file Table_2.DOCX]

**Supplementary data Table 2. Effects of BMP2 on the developmental potential of 2-cell mouse**

**embryos.**

48 h 72 h

No. of blastocyst No. of blastocyst No. of blastocyst

(%) (%) hatching (%)

Control 110^a^ 51 (46.4) 95 (86.4) 53 (48.2)

BMP2 111^a^ 74 (66.7)* 101 (91) 79 (71.2)*

*P* value 0.002 0.278 0.002

^a^ Number of embryos that were examined.

*P<0.05 compared with Control.
